# Readmission of adolescent psychiatric patients to a specialised unit in Gauteng, South Africa

**DOI:** 10.4102/sajpsychiatry.v29i0.2018

**Published:** 2023-07-27

**Authors:** Sarah-Anne Brown, Tshepiso D. Moeketsi, Alfred Musekiwa, Saiendhra V. Moodley

**Affiliations:** 1 Faculty of Health Sciences, University of Pretoria, Pretoria, South Africa; 2 Department of Psychiatry, Faculty of Health Sciences, University of Pretoria, Pretoria, South Africa; 3 School of Health Systems and Public Health, Faculty of Health Sciences, University of Pretoria, Pretoria, South Africa

**Keywords:** psychiatry, South Africa, adolescent, adolescent psychiatry, readmission, readmission factors, readmission rate, Gauteng

## Abstract

**Background:**

Readmission rates to child and adolescent psychiatric units among the youth have been reported to be increasing.

**Aim:**

The study aimed to determine the readmission rate and factors associated with readmission of adolescent psychiatric patients at a child and adolescent psychiatric unit.

**Setting:**

A specialised psychiatric hospital in Gauteng province, South Africa.

**Methods:**

This retrospective cohort study utilised data from the records of patients admitted to the adolescent inpatient unit over a period of five years. The cumulative incidence and incidence rate of readmission within one year of discharge from the index admission was calculated using survival analysis methods. Characteristics significantly associated with readmission were determined by applying the multivariable Cox proportional hazards regression model.

**Results:**

Among the 189 patients included in the analysis, the cumulative incidence of readmission within one year of discharge was 17.5%. The incidence rate was 5.31 readmissions per 10 000 person-days. The final multivariable model showed that a diagnosis of schizophrenia (*p* = 0.015), a diagnosis of attention deficit hyperactivity disorder (*p* = 0.039), and coming from a child and youth care centre or temporary safe care (*p* = 0.018) increased the risk of readmission while having a medical condition (*p* = 0.008) reduced the risk.

**Conclusion:**

Psychiatric diagnosis and residential care could be potential risk markers for readmission. Improving the collaboration between health and social services in residential care would be beneficial.

**Contribution:**

Identifying factors that predispose adolescent psychiatric patients to readmission can inform and improve management and risk assessments.

## Introduction

Worldwide, it has been reported that 10% – 20% of children and adolescents will experience a mental disorder with a majority of these individuals not seeking or receiving care.^1^ It has been reported that 18.5% of South Africa’s population are adolescents, estimated at over 10 million individuals.^2^ Although data are not readily available on prevalence rates of mental health in South Africa, one study found an overall prevalence of 17% for children and adolescents.^3^ High levels of depression and post-traumatic stress symptoms have been found in adolescents from South Africa’s largest city of Johannesburg.^4^

In the United States of America, 15% of all paediatric hospitalisations were attributed to psychiatric hospitalisations.^5^ Previous studies report the incidences of readmission in adolescent psychiatric patients to be between 12% and 37%.^6,7^ Since the onset of managed care, readmission rates for psychiatric hospitalised youth have increased with studies indicating that 33% – 38% will be readmitted within 1 year of discharge.^8^ A systematic review and meta-analysis on readmission among children and adolescents found that 13.2% of adolescents were readmitted.^9^ A study done in the Western Cape province, South Africa, found the rate of readmission in adolescents to be 36%.^10^ The rates of repeated admission to psychiatric inpatient care among the youth are increasing and the risk of rehospitalisation after psychiatric inpatient treatment is becoming more frequent.^11,12^

Little is known about the predictors of readmission among adolescent psychiatric patients.^11,13,14,15,16^ While there is a lot of literature focusing on adult readmission, there are relatively fewer studies and less knowledge about factors that predispose adolescents to readmission. Previous studies reported inconsistent findings regarding the effects of demographic characteristics on the readmission of adolescents. Based on previous research, it is also difficult to draw definitive conclusions as different diagnostic criteria, factors and methodologies were used in different studies.^9^

Of the research that has been done internationally, common findings have found suicidality at index admission^16,17,18,19^ as well as discharge to residential treatment to be risk factors for readmission of adolescent psychiatric patients.^12,20,21,22^ A history of sexual abuse has also been found in some studies to have an association with readmission.^11,14^ Prior hospitalisation history has been found to result in a higher risk of readmission in the adolescent patient.^6,7,10,14,19,23^ Patients with more severe psychotic disturbances and psychotic disorders were found at a significantly higher risk of readmission compared to those without a psychotic disorder.^10,12,19,23,24,25,26,27^

The research in this area in South Africa is very limited. A study done by Pieterse et al.^10^ in the Western Cape province examined the risk factors associated with adolescent psychiatric readmission. The study found that prior admission and older age were associated with readmission of adolescent psychiatric patients.^10^ Furthermore, lower grade of schooling and dropping out of school were associated with readmission, making schooling a protective factor against readmission.^10^

Identifying risk factors that predispose adolescent psychiatric patients to readmission will assist health care practitioners to provide better care, treatment and rehabilitation for the patients and further assist to implement better risk assessment tools and strategies to lower readmission rates among the adolescent psychiatric patients. The study aimed to determine the cumulative incidence and incidence rate of readmission of adolescent patients to a child and adolescent psychiatric unit in the Gauteng province of South Africa and to determine the factors associated with readmission of adolescent inpatients.

## Research methods and design

### Study design

This study was a retrospective cohort study using patient records. The clinical files of all adolescent patients who were admitted during the period 01 January 2013 to 31 December 2017 to the hospital were reviewed. The patient’s index admission and discharge dates were recorded, and information was gathered for multiple ‘exposures’ (potential risk factors) at the time of index admission. These records for each patient were then reviewed for a period of 1 year post-discharge from their index admission to ascertain if they were readmitted or not in this period.

### Study setting

This study was conducted at a public sector specialised psychiatric hospital situated in Gauteng province, South Africa. The hospital has a child and adolescent unit which caters for children who are younger than 13 years and adolescents who are between the ages of 13 and 18 years.

### Study population and sampling strategy

The study population were adolescent patients admitted to the hospital during the period of January 2013 to December 2017. Only patients over the age of 13 years and under the age of 18 years were included in the study. Patients admitted directly to the hospital as well as those who were initially admitted at a different facility and then transferred to the hospital for continuation of care, treatment and rehabilitation were included in the study. There was no sampling as all patients who met the inclusion criteria (namely adolescent patients admitted to the unit between the ages of 13 and 18 years from January 2013 to December 2017) were included in the study.

### Measurements and data collection

Data were collected from clinical files of the adolescent patients using a structured data capture sheet. These files contained documents including admission forms, progress forms, prescription forms, consultation notes and supporting documents such as affidavits or school reports. These files also contained notes from other health care workers including nurse’s reports, psychologist notes and social care worker involvement. The date of admission and discharge for their index inpatient stay was captured as well as demographic information, psychiatric diagnosis and social history (comprising of substance use, physical abuse, emotional abuse and sexual abuse) at the time of the index admission. The date of their first readmission following that discharge was recorded if the patient was readmitted within 1 year of discharge from the index admission.

### Statistical methods

The data were captured in Microsoft Excel and exported to Stata version 16 for analysis.^28,29^ Frequencies and proportions were calculated for the demographic characteristics of the participants. Kaplan-Meier survival analysis was performed.^30^ The cumulative incidence was calculated by dividing the number of patients readmitted within 365 days of discharge from their index admission by the total number of patients in the study population that were discharged from their index admission. The incidence rate of readmission was calculated by dividing the number of patients readmitted within 365 days of discharge from their index admission by the total number of person-days at risk. Person-days at risk was calculated by adding the number of days to readmission or 365 days for those that were not readmitted.

Demographic and clinical characteristics associated with readmission within 1 year of discharge were determined using the Cox proportional hazards regression model. All variables that were found to have *p* < 0.25 on univariate Cox regression model were included in a multivariable Cox regression model. A manual backward elimination process was used to arrive at a final model. Results are displayed using a table showing the unadjusted and adjusted hazard ratios with their corresponding 95% confidence intervals and *p*-values. A *p*-value ≤ 0.05 was considered statistically significant. Individuals with missing data on either outcome or risk factor were excluded from analysis, and therefore a complete case analysis was performed. No data imputation was performed, and the statisticians assumed the data to be missing completely at random.

### Ethical considerations

Ethical approval was granted for this study by the Faculty of Health Sciences Research Ethics Committee of the University of Pretoria (278/2019). Permission was obtained to collect data from the psychiatric hospital from its Chief Executive Officer. The names of the patients were not included on the data sheet, with a unique study number allocated to each patient instead to ensure anonymity of the patients. A separate electronic spreadsheet was kept with the names of the patients and their unique study number to prevent double counting of records and to ensure files could be accessed in the event of a data query; this was deleted immediately after the statistical analysis was conducted. Patient confidentiality was maintained throughout the research process.

## Results

**FIGURE 1:**
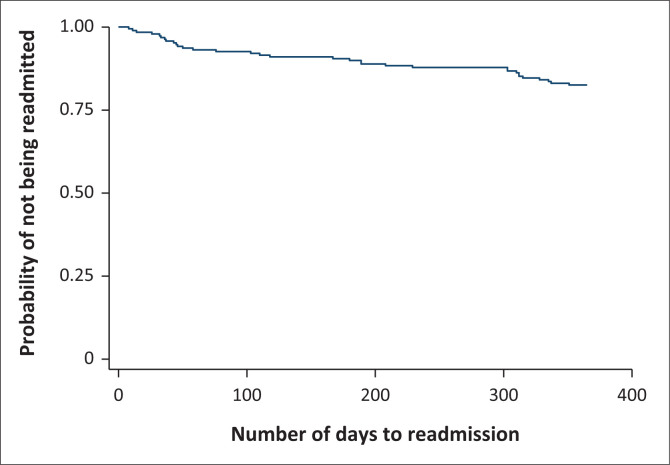
Kaplan-Meier survival estimate of adolescent patients being readmitted to the psychiatric hospital within one year of discharge.

In total, the records for 212 patients were reviewed. However, only records for 189 patients were included in the analysis. Those excluded did not meet the inclusion criteria, did not have the required data for almost all variables or did not have a discharge date that could be ascertained. Of the 189 patients, 33 were admitted within 1 year of discharge, which is a cumulative incidence of 17.5%. The incidence rate was 5.31 readmissions per 10 000 person-days (33/62154 days). The median time to readmission for the 33 patients that were readmitted within 1 year of discharge was 118 days (range = 8–351 days). Of these 33 patients readmitted within 1 year of discharge, four patients (12.1%) were readmitted within 1 month of discharge, 15 patients (45.6%) were readmitted 1 to 6 months post-discharge and 14 patients (42.4%) were readmitted more than 6 months post-discharge. The Kaplan-Meier graph is shown in Figure 1.

The demographic characteristics of the patients in the study at the time of their index admission are shown in Table 1. More than 40% of patients in the study were between 15 and 16 years of age. There were more male (53.4%) than female patients (46.6%). Most patients were in grade 8–12. Most patients were from an urban area with only four patients (2.2%) from a rural area. The most common primary caregiver was a combination of a biological parent and nonbiological parent (23.0%). More than 10% of patients were from a child and youth care centre or temporary safe care.

**TABLE 1:** Demographic characteristics of adolescent patients at index admission to the psychiatric hospital.

Characteristic (*N*)	*n*	%
**Age (*N* = 189)**		
13–14 years	47	24.9
15–16 years	81	42.9
17–18 years	61	32.3
**Gender (*N* = 189)**		
Female	88	46.6
Male	101	53.4
**Highest level of education (*N* = 182)**		
Special school	14	7.7
Grade 1–4	2	1.1
Grade 5–7	32	17.6
Grade 8–12	134	73.6
**Type of residential area (*N* = 186)**		
City or town (including suburbs)	137	73.7
Township	44	23.7
Rural	4	2.2
Other	1	0.5
**Primary caregiver (*N* = 187)**		
Biological parents still married	27	14.4
Biological parent and nonbiological parent	43	23.0
Single parent previously married	34	18.2
Single parent never married	28	15.0
Adoptive parents	11	5.9
child and youth care centre or temporary safe care	21	11.2
Other	23	12.3

The unadjusted hazard ratios for readmission within 1 year of discharge and demographic characteristics at index admission are shown in Table 2. The only unadjusted hazard ratio with respect to the demographic variables that was statistically significant was primary caregiver, specifically patients from child and youth care centre or temporary safe care were at a higher risk for readmission (HR 10.84, 95% CI 1.33–88.16, *p* = 0.026) when compared to biological parents that were still married.

**TABLE 2:** Unadjusted hazard ratios for readmission of adolescent patients within 1 year of discharge from the psychiatric hospital and demographic characteristics at their index admission (univariate Cox regression).

Characteristic	Hazard ratio	95% confidence interval	*p*-value
**Age in years (*N* = 189)**			
13–14	1.00	-	-
15–16	1.03	0.43–2.45	0.953
17–18	1.09	0.44–2.70	0.860
**Gender (*N* = 189)**			
Male	1.00	-	-
Female	0.62	0.30–1.25	0.182
**Type of residential area (*N* = 181†)**			
Township	1.00	-	-
City or town including suburbs	0.84	0.39–1.81	0.663
**Highest level of education (*N* = 182)**			
Grade 1–7	1.00	-	-
Grade 8–12	1.07	0.44–2.62	0.875
Special school	0.37	0.04–3.09	0.360
**Primary caregiver (*N* = 187)**			
Biological parents still married	1.00	-	-
Biological parent and nonbiological parent	6.05	0.77–47.77	0.088
Single parent previously married	3.38	0.38–30.28	0.276
Single parent never married	5.52	0.64–47.25	0.119
Adoptive parents	8.09	0.84–77.77	0.070
Child and youth care centre or temporary safe care	10.84	1.33–88.16	0.026
Other	5.11	0.57–45.76	0.144

† , ‘Rural’ and ‘Other’ excluded due to insufficient data.

The unadjusted hazard ratios for readmission within 1 year of discharge and clinical characteristics at index admission are shown in Table 3. The unadjusted hazard ratios with respect to clinical characteristics that were statistically significant were presence of medical condition(s), a diagnosis of attention deficit hyperactivity disorder (ADHD), a diagnosis of post-traumatic stress disorder (PTSD) and presence of multiple psychiatric disorders. Patients with one or more medical conditions were at a lower risk for readmission (HR 0.41, 95% CI 0.18–0.95, *p* = 0.039) than those without a medical condition. Compared to patients with other diagnoses, those with ADHD (HR 2.37, 95% CI 1.03–5.46, *p* = 0.043) and PTSD (HR 4.19, 95% CI 1.00–17.55, *p* = 0.050) were at higher risk of readmission. Patients with multiple psychiatric disorders (HR 2.16, 95% CI 1.08–4.31, *p* = 0.029) were more likely to be readmitted than those with a single disorder.

**TABLE 3:** Unadjusted hazard ratios for readmission of adolescent patients within 1 year of discharge from the psychiatric hospital and clinical characteristics at their index admission (univariate Cox regression).

Characteristic	Hazard ratio	95% confidence interval	*p*-value
**Patient history**			
Previous Weskoppies Hospital admission	1.28	0.49–3.32	0.610
Suspected abuse (*N* = 153)	0.92	0.43–1.97	0.827
Confirmed abuse (*N* = 133)	1.00	0.45–2.19	0.991
Social worker involvement (*N* = 165)	1.94	0.83–4.49	0.124
Criminal history (*N* = 132)	1.78	0.75–4.20	0.191
Substance use (*N* = 172)	0.64	0.30–1.37	0.253
Family upbringing problems (*N* = 172)	1.22	0.37–4.02	0.738
Medical condition(s) (*N* = 180)	0.41	0.18–0.95	0.039
Family history of psychiatric disorders (*N* = 114)	0.90	0.36–2.22	0.814
**Diagnoses**			
Intellectual disability (*N* = 186)	0.44	0.06–3.19	0.414
Communication disorders (*N* = 186)	Insufficient data	-	-
Autism (*N* = 186)	Insufficient data	-	-
Attention deficit hyperactivity disorder (*N* = 186)	2.37	1.03–5.46	0.043
Specific learning disorders (*N* = 186)	2.23	0.30–16.35	0.429
Mood disorders (*N* = 186)	0.96	0.48–1.94	0.919
Schizophrenia (*N* = 186)	1.89	0.90–3.98	0.092
Bipolar disorder (*N* = 186)	0.55	0.23–1.33	0.183
Anxiety disorders (*N* = 186)	3.11	0.74–13.04	0.120
Post-traumatic stress disorder (*N* = 186)	4.19	1.00–17.55	0.050
Elimination disorders (*N* = 186)	Insufficient data	-	-
Feeding disorders (*N* = 186)	Insufficient data	-	-
Disruptive disorders (*N* = 186)	1.72	0.77–3.80	0.184
Substance disorders (*N* = 186)	1.17	0.51–2.70	0.711
Neurocognitive disorders (*N* = 186)	Insufficient data	-	-
Other disorders (*N* = 186)	0.48	0.07–3.53	0.473
Multiple psychiatric disorders (*N* = 186)	2.16	1.08–4.31	0.029
**Treatment (*N* = 187)**			
Psychotropic medication	1.00	-	-
Psychotherapy	8.11	0.51–129.72	0.139
Both psychotropic medication and psychotherapy	5.06	0.69–37.07	0.111

The initial multivariable Cox regression model consisted of gender, primary caregiver, social worker involvement, criminal history, presence of medical condition(s), diagnosis of ADHD, diagnosis of schizophrenia, diagnosis of bipolar disorder, diagnosis of an anxiety disorder, diagnosis of PTSD, diagnosis of disruptive disorder, presence of multiple psychiatric disorders and treatment. Following backward elimination, the final model consisted of child and youth care centre or temporary safe care as the primary caregiver, presence of a medical condition, diagnosis of ADHD and diagnosis of schizophrenia (see Table 4). Patients from child and youth care centres or temporary safe care (HR 2.99, 95% CI 1.21–7.42, *p* = 0.018), patients with a diagnosis of schizophrenia (HR 2.68, 95% CI 1.21–5.94, *p* = 0.015) and patients with a diagnosis of ADHD (HR 2.68, 95% CI 1.05–6.85, *p* = 0.039) were significantly more likely to be readmitted within 1 year of discharge, while patients who had one or more medical conditions (HR 0.32, 95% CI 0.13–0.74, *p* = 0.008) were significantly less likely to be readmitted within 1 year of discharge.

**TABLE 4:** Multivariate Cox regression model for readmission of adolescent psychiatric patients to the psychiatric hospital within 1 year of discharge (*N* = 179) from their index admission.

Characteristic	Hazard ratio	95% confidence interval	*p*-value
Child and youth care centre or temporary safe care	2.99	1.21–7.42	0.018
Medical condition(s)	0.32	0.13–0.74	0.008
Attention deficit hyperactivity disorder	2.68	1.05–6.85	0.039
Schizophrenia	2.68	1.21–5.94	0.015

## Discussion

Our study aimed to determine readmission rates and factors that predispose adolescent psychiatric patients to readmission at a specialised psychiatric hospital in the Gauteng Province, South Africa. The readmission cumulative incidence found in this study (17.5%) seems to be similar to other studies (12%–37%) done elsewhere.^6,7,10,11,13,14,15,16,31,32,33^ The wide range of readmission incidences among previous studies could be due to different methodologies and definitions. Multivariable Cox regression model was used to identify the factors significantly associated with readmission within 1 year of discharge. The factors identified in this study with an increased risk of readmission were adolescents coming from a child and youth care centre or temporary safe care, a diagnosis of ADHD or a diagnosis of schizophrenia while having a medical condition seemed to decrease the risk for readmission.

Our finding of schizophrenia predisposing adolescent psychiatric patients to readmission has been found in previous studies.^7,23,24,26,27,34,35,36^ Schizophrenia is known to be chronic in nature and has shown to have a poorer prognosis in comparison to other mental illnesses.^25,32,37^ Schizophrenia presents with symptomatology which is difficult to treat; psychotic symptoms have been found to require longer acute treatment and are more likely to be treated in a hospital setting.^24,26^ Adolescent onset schizophrenia has been found to have a poorer prognosis than adult-onset psychosis with less chance of full recovery; only a minority of youths display a complete recovery.^37,38,39^ Due to the severity of the illness and complex treatment, this is shown as a contribution to this diagnosis being associated with readmission.

Another explanation of this finding could be linked to poor aftercare of the schizophrenia adolescent. It has been shown that caregivers report difficulty to care for a mentally ill adolescent.^40,41^ Adequate aftercare with good follow-up compliance has shown to have a positive correlation with lower readmission rates in adults.^42^ Because most district and regional hospitals in South Africa cannot manage the severity of schizophrenia, most of these patients are referred to tertiary units. These referrals, instead of receiving adequate management at these hospitals, may contribute to the high readmission rate.

The study suggests that having the diagnosis of ADHD is associated with readmission to a psychiatric hospital. In this study, all readmitted adolescents diagnosed with ADHD had other psychiatric comorbidities with the most common comorbidity being disruptive and/or conduct disorder followed by bipolar disorder. Studies have found a strong correlation between readmission and bipolar disorder in adolescents.^8,10,12,35,43,44^ However, we did not find bipolar disorder to be an independent risk factor for readmission. A complex burden of psychiatric comorbidities in adolescents diagnosed with ADHD may predispose these adolescents to being readmitted to a psychiatric hospital.

This study found that coming from a child and youth care centre or temporary safe care^45^ predisposes adolescents to readmission to a psychiatric hospital. Although the literature has noted the importance of mental health service needs among the youth care centre and temporary safe care population, the high workload and social worker crisis in South Africa seems to result in social workers focusing more on the physical needs of child and youth care centre and temporary safe care youth while neglecting more of their psychosocial needs.^46^

Youth in a child and youth care centre or temporary safe care would be more vulnerable to not receiving care due to lacking a person in their life who is responsible and accountable for them.^47^ One study found that youth in a child and youth care centre or temporary safe care have an increased risk of not receiving services necessary for their needs; this could be due to financial costs and poor health care systems.^47^ Children in residential care need safety and supportive relationships with direct care staff as it is a critical element to recovery, growth and development.

A study found mental health service utilisation in this population to be poor; much of child and youth care or temporary safe care was found to be delivered without significant mental health services for children.^48^ Little research is available with regard to the mental health needs of child and youth care or temporary safe care children in South Africa. Further research is needed to establish the mental health service accessibility to child and youth care or temporary safe care. Poor compliance to treatment, especially outpatient treatment, could be a possible reason for readmission in the adolescent population.

This study found that adolescent patients with a comorbid medical condition were less likely to be admitted. This finding has not been reported in the literature previously and would benefit from exploration in future research. Possible explanations of this finding could be that the presenting psychiatric condition may have been a psychological reaction to a newly diagnosed medical condition which could have led to a temporary or brief psychiatric disorder or it could have been a complication of the medical condition so that appropriate management of the medical condition prevented further readmissions. Furthermore, a diagnosis of a medical condition could allow the patient to have potentially better and more frequent access to medical care. This could further assist in addressing comorbid psychiatric problems in other clinical settings, thereby avoiding admission or readmission to psychiatric hospitals. Future research is needed when examining treatment factors and community-level factors with regard to readmission.

### Limitations

This study was a retrospective review of clinical files which has limitations with respect to data availability. Patient history, illegible handwriting, missing information, availability of collateral information and follow-up information all negatively affected the available data. A few patients could not be included due to missing data in their record for the key variables.

As we used the definition of readmission as readmission within a 1 year period post-discharge from the index admission, we did not collect data and cannot report on readmissions that may have occurred after that time period. Readmissions to other psychiatric hospitals were also not taken into consideration and we only looked at readmissions of the patient during their adolescent years, meaning that if a readmission were to happen 1 year out of the designated age, this too would not be counted.

## Conclusion

Approximately 17.5% of adolescent psychiatric patients at a specialised psychiatric hospital in the Gauteng province of South Africa were readmitted within 1 year of discharge. A diagnosis of schizophrenia, ADHD and coming from a child and youth care centre or temporary safe care increased the risk of readmission while having a medical condition appeared to reduce the risk of readmission. A lack of aftercare services, social development services, adequate hospital care and mental health care services for adolescent psychiatric patients may contribute to readmission of these patients. Improved collaboration between the Department of Health and Department of Social Development is needed to improve and ensure management of adolescent psychiatric patients and to develop policies that are aimed at reducing readmission of these patients into psychiatric hospitals.
